# Current diagnostic procedures for diagnosing vertigo and dizziness

**DOI:** 10.3205/cto000141

**Published:** 2017-12-18

**Authors:** Leif Erik Walther

**Affiliations:** 1 HNO-Gemeinschaftspraxis, Main-Taunus-Zentrum, Sulzbach, Germany

**Keywords:** dizziness, vertigo, video head impulse test, VEMP, cVEMP, oVEMP

## Abstract

Vertigo is a multisensory syndrome that otolaryngologists are confronted with every day. With regard to the complex functions of the sense of orientation, vertigo is considered today as a disorder of the sense of direction, a disturbed spatial perception of the body. Beside the frequent classical syndromes for which vertigo is the leading symptom (e.g. positional vertigo, vestibular neuritis, Menière’s disease), vertigo may occur as main or accompanying symptom of a multitude of ENT-related diseases involving the inner ear. It also concerns for example acute and chronic viral or bacterial infections of the ear with serous or bacterial labyrinthitis, disorders due to injury (e.g. barotrauma, fracture of the oto-base, contusion of the labyrinth), chronic-inflammatory bone processes as well as inner ear affections in the perioperative course. In the last years, diagnostics of vertigo have experienced a paradigm shift due to new diagnostic possibilities. In the diagnostics of emergency cases, peripheral and central disorders of vertigo (acute vestibular syndrome) may be differentiated with simple algorithms. The introduction of modern vestibular test procedures (video head impulse test, vestibular evoked myogenic potentials) in the clinical practice led to new diagnostic options that for the first time allow a complex objective assessment of all components of the vestibular organ with relatively low effort. Combined with established methods, a frequency-specific assessment of the function of vestibular reflexes is possible. New classifications allow a clinically better differentiation of vertigo syndromes. Modern radiological procedures such as for example intratympanic gadolinium application for Menière’s disease with visualization of an endolymphatic hydrops also influence current medical standards. Recent methodical developments significantly contributed to the possibilities that nowadays vertigo can be better and more quickly clarified in particular in otolaryngology.

## 1 Introduction

In medicine, the interdisciplinary symptom of vertigo represents a particular challenge [1]. It is one of the most frequently observed leading symptoms [2], [3].

Episodic or permanent vertigo impair the quality of life as well as the independence and self-determination of movement. The complexity of the impairment by vertigo affects all parts of daily life. Persisting complaints promote anxiety and may lead to the development of depression. Permanent vertigo may further be associated with a reduced physical activity, loss of social contacts up to possible inability to work. If a tendency to fall occurs, severe complications and care dependency in higher ages may result [4], [5], [6].

Diagnosing vertigo syndromes is particularly complex since the causes for the perception of vertigo are manifold, and it is often difficult to find the correct diagnosis. So the treatment of vertigo syndromes requires an experienced diagnostic approach. Preconditions are special and specific knowledge, clinical experience, interdisciplinary cooperation, updated education and training, and continuous learning [7].

Over the last years, the diagnosis of vertigo has progressed enormously [7] [8], [9], [10], [11], [12], [13], [14], [15], [16], [17], [18], [19], [20], [21]. Knowledge about vestibular reflex structures [22], [23], [24], [25] led to the development of new diagnostic approaches that could be implemented rapidly in clinical practice due to technical progress. Some of those new diagnostic procedures are the clinical head impulse test, the video head impulse test (vHIT) [26], [27], [28], [29], [30], [31], the assessment of the visual acuity (dynamic visual acuity, DVA) [32], and the cervical and ocular vestibular evoked potentials (VEMP) [23], [24], [25], [33], [34], [35]. For the first time, the function of vestibular reflexes can be measured with those modern methods, completed by conventional diagnostics (e.g. caloric testing, rotational tests) in a complex, objective, side-specific, quantitative, and mainly receptor- and reflex-specific way. The function of the vestibular reflexes cannot only be assessed in a differentiated way, i.e. with regard to topology, but the knowledge that the sense of direction – and especially the vestibulo-ocular reflex (VOR) – works in a broad frequency range (frequency dynamics) led to a new understanding of the outcome of physiological test results [36], [37], [38], [39], [40], [41]. Topological and frequency-specific analyses as well as the assessment of changes in the time course (timely dynamics) were summarized in the concept of a differentiated vestibular functional analysis [37].

The development of those modern diagnostic instruments led to a paradigm shift in clinical vestibular diagnostics that increasingly influences the current medical standards of the diagnosis of vertigo and balance disorders [37]. In Germany, modern methods are more and more established in the outpatient and inpatient ENT-specific sector [37], [38], [42], [43], [44], [45]. The medical benefit of modern diagnostic procedures, their quality and effectiveness are meanwhile scientifically confirmed. Diseases may be identified more reliably and rapidly, they may be differentiated and classified more easily and thus be treated in a more effective and swifter way. Today, modern diagnostic procedures allow a precise differentiation of acute unilateral vestibulopathy from stroke with vestibular symptoms, for example by applying the video head impulse test [46], [47], [48], [49]. Measurement and quantification allow the exact control of therapeutic interventions at the vestibular organ as for example the intratympanic application of gentamicin in the context of Menière’s disease or after surgical interventions at the labyrinth [50], [51], [52], [53], [54], [55]. Also the diagnostic approach in the context of medical reports concerning vertigo is determined by modern diagnostic procedures [56].

Under a practical point of view, this article will summarize and critically discuss current aspects of modern vertigo diagnostics.

**Conclusion:** Vertigo is a subjective symptom. By means of modern diagnostic concepts with high reliability, exhaustive objective clarification of the question is possible if the complaints have a vestibular cause or not.

## 2 Diagnostic basics in the context of vertigo

Over the last years, a standardized vocabulary for diseases with the leading symptom of vertigo was established in the literature. Vertigo has a syndrome character; it dominates subjectively as perception, but individually it appears in very different ways, for example in combination with vegetative concomitant events, disturbed spatial orientation, impairment of posture (tendency to fall) and gait (unsteady gait) as well as psychic symptoms. So diseases with the main symptom of vertigo are called “vestibular syndromes” (see also ICD-10 H81 and H82, version of 2016) [2], [3], [57], [58], [59].

A classification of vestibular syndromes is mainly based on topology-specific causes. According to the location of the origin, vestibular syndromes are classified for didactic reasons and for topologic differentiation into peripheral vestibulopathy and central vestibulopathy [3], [59], [60]. Regarding the psychogenic origin and involvement, different terms are used in the literature (primary and secondary somatoform vertigo, phobic staggering vertigo, chronic subjective vertigo) based on the cause [61], [62], [63], [64]. Recently, efforts were made to summarize them as functional vertigo syndromes [59], [65]. Causes concerning internal medicine (e.g. orthostatic hypotension, syncopes, cardiac arrhythmia) where vestibular reflexes are not causally involved are not included in the term of vestibular vertigo [66]. Rarer origins in the ophthalmological sector (e.g. refraction problems) are often described as ocular or ophthalmological vertigo [67]. There are controversies regarding cervical or cervicogenic vertigo [68], [69]; in the acute stage, in general its existence is no longer doubted [70].

The orientation at separate medical disciplines plays a major role in the context of competences of interdisciplinary cooperation, but also of medical reporting. The basic reference hereby is always the current version of the education rules and regulations of the German Medical Association (Bundesärztekammer).

According to the duration of the complaints, the difference is made between acute and chronic vertigo syndromes. Vertigo syndromes with symptom-free intervals are called episodic (paroxysmal) vertigo. They can be differentiated against permanent complaints (permanent vertigo) [66], [71].

The acute phase with vertigo is called acute vestibular syndrome. For unilateral acute peripheral disorders, recently the term of “acute unilateral vestibulopathy” was coined [72] (e.g. vestibular neuritis). Differential diagnostic overlapping exists with regard to brainstem or cerebellar infarction (acute central vertigo) that may present with nearly identical symptoms of acute unilateral peripheral vestibulopathy [3].

Vertigo syndromes lasting for several weeks or months are classified as chronic vertigo syndromes. The causes may be peripheral vestibulopathies with insufficient vestibular compensation or functional vertigo.

Gait disorders are often associated with vertigo [73], [74]. The movement by walking is a daily-life performance with natural sensorimotor control requiring the complicated interaction of motor skills, sensory control, and cognitive function. In higher ages or because of diseases, those sensorimotor skills are often impaired. Age-associated gait disorders summarize a disturbed qualitative and quantitative movement process, a reduction of the speed, and impaired gait initiation and control of the body balance [73], [74]. Frequent causes are sensory deficits (bilateral vestibulopathy, polyneuropathy), degenerative diseases (Parkinson’s disease), or toxic influences (alcohol) [73], [74].

A defined vocabulary is used in vestibular diagnostics. The vestibulo-ocular reflex, the neuronal connection between the semicircular canals and the eye muscles, can be conventionally analyzed by caloric stimulation of the horizontal semicircular canal (horizontal VOR, hVOR) that objectively assesses the low-frequency range of the VOR and the lateral semicircular canals [1], [2]. The head impulse test (HIT) reflects the vestibulo-ocular reflex in the high-frequency range [37]. The test that is not device-related is called the clinical HIT. The technical test is the video head impulse test (video HIT, vHIT) [38], [39]. Main components are very light video goggles that are connected with a computer (laptop). In this way, the VOR may be displayed objectively. By means of the vHIT, the VOR of all semicircular canals can be analyzed quantitatively and selectively. The vHIT is the only method to objectively analyze the VOR of the superior semicircular canal.

The identification of the otolith-related reflex pathways has also created the possibility to objectively diagnose selected otolith organs [23], [24], [25]. Sacculo-collic and utriculo-ocular reflexes can be measured based on vestibular evoked myogenic potentials (VEMP) in air conduction or in single cases in bone conduction by means of surface myography [33], [34]. The cervical measurements (cVEMP) (about <500 µV) in air conduction reflect the dominating sacculus function or the sacculo-collic reflex. Ocular air-conduction-induced VEMP (oVEMP) (about <20 µV) are an indicator for the dominant percentage of the otolith-ocular reflex or the utricular function [42], [43], [44], [45]. The part of the reflexes for cVEMP and oVEMP stimulation was recently calculated by Govender et al. [75]. Until then, there was a passionate controversy in the literature about the oVEMP diagnostics [76], [77], [78], [79] that seems to be finished now. Strictly speaking, VEMP diagnostics consist of extrapolating the otolith function on the acoustically sensitive parastriolar type 1 hair cells of the sacculus and utriculus that have a frequency range to be stimulated of about <100 to >4 kHz. The optimal stimulation frequency in air conduction amounts to 500 Hz; this frequency is currently mostly used in practice.

Research results of the last years could show that there are important interactions between vestibular-visual and somato-sensory functions. Vestibular functions also influence central processes such as mental processes, spatial memory, and navigation [80], [81], [82], [83]. Those processes, summarized recently in the concept on higher vestibular functions, which might appear for example in the context of peripheral vestibulopathies with symptoms of disturbed multisensory integration, require among others a detailed analysis of cognitive functions [84]. So vertigo must be considered as a complex impairment of the multisensory functions of the sense of direction.

For some peripheral vestibulopathies (e.g. benign paroxysmal positional vertigo, Menière’s disease) and for vestibular migraine, recently new recommendations have been elaborated [85], [86], [87], [88]. They are mainly based on clinical aspects. The classification of Menière’s disease represents a consensus of international societies under the coordination of the Bárány Society for Neuro-Otology in cooperation with the American Academy of Otolaryngology, Head & Neck Surgery (AAO-HNS) [85], [86], [87], [88]. The AAO-HNS criteria developed in 1995 were thus revised [89], [90].

In 2006, Bisdorff et al. presented an international classification of vestibular diseases (International Classification of Vestibular Disorders, ICVD) [91]. 

The guidelines on vertigo (separate guidelines for diagnostics and therapy) were established by the German Society of Neurology in 2012 and were applied until 2015 [92]. Finalization of the revised guideline on vestibular functional disorders coordinated by the German Society of Oto-Rhino-Laryngology, Head & Neck Surgery (application dated April 29, 2016) in cooperation with the German Society of Neurology is expected for December 31, 2017 [93].

**Conclusion:** The treatment of vertigo (in the field of neuro-otology) requires special knowledge regarding anatomy, physiology, and pathophysiology of the vestibular system and an interdisciplinary approach. The current concepts in teaching, education, and healthcare do not sufficiently take into account the interdisciplinary aspect.

## 3 Vertigo and sense of direction

In order to understand the problem of vertigo, it is necessary that physicians dispose of complex knowledge about the sense of direction. A healthy human organism is able to orient itself spatially and timely at rest and during movement without any problem. Visual (eyes), proprioceptive (skin, muscles, joints) and vestibular senses are the “main entrances” of the sense of direction [8], [37]. Efforts such as an undisturbed gait as well as stabilization of the visual axis under different conditions are continuously required in daily life. This complicated interaction of the different senses is strongly demanded in particular during movement, which requires an undisturbed perception, transmission, and processing of vestibular, visual, and proprioceptive information [2], [8], [37].

The movement of the body (navigation) presupposes intact sensory entrances (vestibular receptors) and reflective connections (vestibular reflexes), an undisturbed upright posture, and spatial movement (postural control) as well as a stable image on the retina (visual stabilization). Those basic performances of the sense of orientation require intact dynamics encompassing a very broad frequency spectrum in order to cope with the requirements of daily life as function of age. Furthermore, additional information (e.g. hearing ability, somato-sensation), an intact cardio-vascular system as well as good mental health are preconditions for the stability of those functions [84].

When the interaction of those different functions is impaired, vertigo develops. Straumann defines vertigo from a pathophysiological point of view as disorder of the sense of direction, a disturbed perception of the spatial position of the body [8].

According to Bisdorff [91], various definitions of vertigo must be differentiated under phenomenological aspects:

- Internal vertigo: the sensation of own movement (illusion of movement) of the body (vertigo); sensations such as rotating, swaying, and tilting.
- External vertigo: visual sensations of moving surroundings (illusory sensations, oscillopsia).
- Dizziness: disturbed perception of the spatial orientation without illusory movement.
- Standing and gait instability: problems with standing, walking, and sitting [94].

With regard to the complexity of available test procedures, recently the focus has been placed on a frequency-specific assessment of the results of test procedures of VOR. It is well-known that the sense of direction works with signals from different frequency ranges in order to assure the extraordinarily high flexibility without any disturbances especially for movement stimuli. The directional movement (navigation) requires low-frequency signals. Higher-frequency signals are necessary for walking and running (about 3–5 Hz). An undisturbed orientation presupposes that steady or moving visual targets can be fixed or pursued visually even if the proper motion of the body (movement of the head and vibration) occur (frequencies up to 10 kHz). Furthermore, distances have to be corrected. During those performances and corrections, a sharp image has to be continuously projected on the retina [8], [37]. Especially the systems of the eye movement (smooth pursuit, saccadic system, vergence system) and VOR [38], [39] contribute to this performance. Because of the short latency of the VOR (about 7–10 ms) and its high dynamic properties, the VOR plays a crucial role for visual stabilization. Physiologically less important low-frequency ranges for the daily function of the VOR that are nonetheless significant aspects for functional diagnostics are assessed by means of caloric tests. Rotatory test procedures and the video head impulse test reflect middle- and high-frequency properties of the VOR. The understanding of those correlations allows interpreting the test results in the context of disorders of the sense of direction [37], [38], [39].

**Conclusion: **Vertigo is a result of an impairment of the sense of orientation. The elements of the sense of orientation work with signals of different frequency ranges. Thus, the extraordinarily high flexibility for movement stimuli can be realized without disturbances. 

## 4 Diagnostic procedure for vertigo

The diagnostic process of the unspecific symptom of vertigo is based on knowledge gain and takes place in a continuous learning process. It draws on information from the patient’s history (anamnesis), clinical (orienting examinations with qualitative result) and technical examinations (quantification with orientation on reference ranges). Specific interdisciplinary information may be added. This process orients on medical standards. At the end of this process, there is the overall assessment (diagnosis) which is often defined according to the degree of certainty with terms like “confirmed”, “probable”, or “possible”. Frequently also terms like “suspected” or “… excluded” are used. 

In the context of vertigo syndromes, it is recommended to refer to the mentioned currently applied classifications [85], [86], [87], [88], [89], [90], [91]. So the mere “possibility” of a diagnosis (e.g. possible Menière’s disease) must be classified as a variant of the reality of low probability. The Bárány Society of Neuro-Otology did no longer include the term of possible Menière’s disease in it revised classification that was still found in the classification of the American Academy, (possibly) because of its low diagnostic probability [86], [87]. In contrast, a clear Menière’s disease speaks for a high probability. This diagnostic probability for vertigo syndromes is significant for the recommendation and introduction of a therapy and its outcome. Vertigo syndromes that do not meet the criteria of current classifications are often described as “atypical” in the scientific literature. This term is an expression of uncertainty or a new entity that has not yet been classified. Even if the classifications are mainly based on anamnestic data, clinical examinations cannot be left out. They contribute to confirming or excluding disorders.

Modern vestibular diagnostics are able to provide objective information. Today it is possible with modern vertigo diagnostic procedures to determine the obvious interrelation between symptoms and diagnoses, which the philosophers Fangerau and Martin called the grade of highest certainty [95]: modern systems of eye movement analysis dispose of video documentation systems and possibilities of optic presentation that allow displaying the examination process in a time interval in a reproducible way. This objective information (also as video) is a convincing “proof” and provides a high degree of evidence. 

In technical vestibular diagnostics, reference ranges are the “measure” for the evaluation of diagnostic results and the criterion for the differentiation between “normal” and “pathological”. The treatment of diseases but also the assessment in the context of ENT-specific expert opinion is based on the results of diagnostic examinations.

The relatively vague term of “gold standard” describes the “best” diagnostic method according to the current state of knowledge and the results of evidence-based medicine that is the reference for modern methods. For example, in the last years, in the context of development of the head impulse test, the question was asked if it might “replace” caloric tests (the current gold standard for the analysis of the hVOR) or if possibly other functions may be assessed. Modern diagnosis of Menière’s disease by means of intratympanic gadolinium application is – according to evaluations performed until now – a “competing procedure” for the current objectification of endolymphatic hydrops by electrocochleography.

The diagnostic procedure for vertigo is not always complicated. In many cases, already targeted questions may lead to specific diagnostic steps in order to find an individual therapy.

**Conclusion:** The diagnostics of vertigo are a continuous learning process ending with an overall assessment (diagnosis). For vertigo syndromes that can be classified, the evaluation of the diagnostic probability is performed according to the degree of certainty. Objective diagnostic procedures have a high degree of evidence.

## 5 History taking for the leading symptom of vertigo

Modern vestibular diagnostics always start with structured history taking. The anamnesis is an important piece in the puzzle of diagnostic assessment. It provides among others information about the type and severity of the complaints, the overall impairment as well as the social and professional setting of the patient. The aim of vestibular diagnostics is the undelayed clarification of the complaints with information of the patient in order to avoid the consequences of balance disorders such as anxiety, secondary somatoform disorders, development of depression, social isolation, and longer absences from work by introducing early therapeutic measures. 

From the patients’ point of view, the symptom is individually interpreted in different but generally similar ways. For the physician, the ambiguous and unspecific symptoms are often difficult to interpret. For retrieving anamnestic information in cases of vertigo, an analytic procedure with the following basic elements in the described sequence has turned out to be useful [66], [71], [94].

The assessment of the severity and the exclusion of severe complications (stroke, syncopes) have high priority (Table 1 (Tab. 1)).

In cases of permanent chronic vestibular complaints, the otolaryngologist has to ask if the complaints are associated with an objectifiable vestibular deficit or not. Since modern diagnostic measures provide the possibility to completely analyze the sensory vestibular functions and reflex pathways (Table 2 (Tab. 2)), the complaints can be attributed with high diagnostic certainty to an objective disorder or in cases of missing hints, disorders of the peripheral functions may be excluded. Frequent causes of chronic complaints are insufficiently compensated acute unilateral vestibulopathies, primary or secondary somatoform disorders or functional vertigo.

Episodic vertigo is characterized by sudden or with aura announced vertigo attacks lasting for some seconds or hours or even days. The differential diagnostic classification may be difficult. The duration of the vertigo attacks and accompanying symptoms (influence of the position, headaches, hearing loss) play a key role for the classification. If no objective symptoms can be assessed, internal and neurological examinations are appropriate. It must also be taken into account that also comorbidities may present in clinical practice. Vestibular migraine for example is often an accompanying symptom of Menière’s disease and other peripheral vestibulopathies. For Menière’s disease, among others, psychic comorbidities are frequently observed [96], [97], [98], [99], [100]. The consultative psychological and psychiatric co-treatment is currently under-represented in Germany.

The diagnostic constellation of vestibular syndromes is different depending on the patient’s age. In higher ages, vertigo (e.g. benign paroxysmal positional vertigo, gait disorders, cardiac arrhythmia, multicausal and multisensory vertigo) is one of the most frequently described complaints. In children and adolescents, the diagnostic constellation includes more rarely observed types of vertigo such as for example vestibular migraine, functional vertigo, or orthostatic dysregulation). In cases of multisensory vertigo, several components (at least 2) of the sense of orientation are impaired. For multicausal vertigo, comorbidities (internal, neurological diseases) must be considered. The anamnestic assessment of falls or near-falls should also be included in history taking. Vertigo is an important risk factor for falls in higher ages. In cases of more than 3 risk factors for falling tendency, a statistically higher risk for falls must be expected [5]. The evaluation of the medication scheme plays a relevant role with regard to the risk of falls. Vertigo and falls are frequently described in the context of application of class 1A antiarrhythmic pharmaceuticals, antihypertensive medication, and psychotropic drugs (so-called FRID; fall risk increasing drugs). The causes of vertigo and falls may also be the side effects or interactions of drugs (cardio-toxic effects with orthostatic or bradycardial reaction) [101], [102], [103], [104]. In daily practice, the current PRISCUS list (potentially inappropriate medication in the elderly) should be applied [105]. If a fall tendency is observed in the context of a vertigo episode, it is also recommended to clarify if a disturbance of consciousness is present. Syncope (e.g. reflex syncope, orthostatic hypotension, cardiac syncope) is defined as suddenly appearing, reversible unconsciousness associated with the loss of postural control. Syncope is rarely associated with the risk of sudden cardiac death. In contrast, the consciousness in the context of loco-motor falls remains intact. Even falls during the so-called Tumarkin’s otolithic crisis (vestibular drop attacks) are experienced with full consciousness [101], [102], [103], [104].

Evaluated German psychometric tests (e.g. Dizziness Handicap Inventory, DHI) further facilitate the classification of the complaints [106], [107]. For classification of the complaints it is also useful when patients keep a “vertigo diary”. 

The evaluation of the information from history taking contributes to planning further diagnostic procedures. After history taking, targeted examinations are performed in cases of pathognomonic indications (e.g. positional maneuvers for benign paroxysmal positional vertigo). In the context of further differentiation, an orienting physical examination of the vestibular and oculo-motor system is recommended.

**Conclusion:** Vertigo should be assessed analytically in the context of history taking. Further diagnostic procedures (clinical examination) can then be performed in a structured way. The causes of acute vertigo have to be clarified with high priority.

## 6 Clinical examination for vertigo syndromes

### 6.1 Orienting examinations for vertigo

Beside the frequently occurring classical vestibular syndromes (e.g. benign paroxysmal positional vertigo, vestibular neuritis, Menière’s disease) with vertigo as leading symptom [2], [3], the otolaryngologist has to bear in mind the multitude of other diseases with the primary or accompanying symptom of vertigo that do not appear in published statistics. Those are hearing losses, auditory canals that are occluded with earwax, disturbed tube function, bacterial (acute otitis media) and viral infections (e.g. influenza-associated otitis, zoster oticus, mumps infection) with labyrinthine association, injury-related disorders (e.g. barotrauma, fracture of the otobase, labyrinthine contusion, rupture of the eardrum), chronic inflammatory bone processes (e.g. chronic epitympanic otitis media), and vertigo after surgical interventions (e.g. open mastoid cavity, stapedotomy, tympanoplasty, cochlear implantation) etc. [1], [55], [69].

So the ENT-specific examination of the symptom of vertigo should start with ear microscopy. In the interdisciplinary context of vestibular examination it plays a key role, especially when “ear symptoms” (e.g. tinnitus, ear secretion, otalgia, hearing loss) are observed. 

Orienting examinations are useful to complete the anamnesis by further limiting the possible causes of vertigo. Without the use of complex devices it is possible to find qualitative symptoms of a disturbed input to the sense of orientation. Examinations of standing and walking, the analysis of eye movement disorders, nystagmus, and oculo-motor functions are in the focus [2], [57], [59], [69].

Posture and gait tests (Romberg’s test, Unterberger test) with open and closed eyes or under challenging conditions (tandem Romberg test, one leg stand) are unspecific but they allow drawing conclusions about problems with standing and walking. Finger tests (finger-nose test, finger-to-finger test) check the coordination [2], [3].

Positional maneuvers are performed among others for anamnestic hints for benign paroxysmal positional vertigo (BPPV) [1], [108]. Beside the German guidelines [92] and the current recommendations of the Bárány Society of Neuro-Otology [85], detailed recommendations of the AAO-HNS [109] and the American Academy of Neurology [110] with evidence-based analyses are available. Most frequently, the posterior semicircular canals and in particular the right posterior semicircular canal are affected [111]. The Dix-Hallpike test (for diagnostics of the posterior semicircular canals) should be at the beginning of the examinations. The objective confirmation before (semicircular canal-) specific nystagmus reaction is considered as proof for the presence of the disease when the classic criteria of peripheral BPPV are fulfilled (short latency, short duration, exhaustibility of the nystagmus, vertigo with vegetative symptoms, directional changes for modified positioning). Canalolithiasis in the short process of the posterior semicircular canal was recently described, however without being able to assign a clear nystagmus reaction (VOR) to this type of vertigo [112]. Overlapping with non-vestibular disorders is possible. Central lesions are a rare cause for positional vertigo [113]. Oculo-motor disorders and cerebellar symptoms are crucial for differential diagnosis. Positional vertigo with or without nystagmus is most often an indication of central disorders.

It is also important to differentiate orthostatic hypotension (changes of blood pressure and heart rate in the context of tilt table tests or Schellong’s test), orthostatic tachycardiac syndrome or bradycardiac arrhythmia. In cases of syncope, undelayed internal diagnostics should be performed in order to find out the causes. Syncope bears the risk of recurrent syncope with sudden heart death.

The correlation between eye position and head and body posture may indicate paresis of the ocular muscles (e.g. paresis of the trochlear nerve). Asymmetry of the head-eye position (ocular tilt) is a symptom complex consisting of vertical squint (Hertwig-Magendie), inclination of the head and eye torsion in cases of peripheral or central disorders.

Eye movement disorders and nystagmus are examined in the 9 gaze positions. The examiner retrieves information about an existing spontaneous nystagmus and the eye motility [114], [115].

Provocation by head shaking at about the level of the horizontal semicircular canal and if needed also nodding may lead to temporary unmasking of unilateral vestibulopathies (about 30 times, 45° amplitude, frequency of 2 Hz). An indication for peripheral vestibulopathy is a horizontal nystagmus under Frenzel goggles induced by provocation in the horizontal plane that goes into the direction of the damaged side in its slow phase. The reason is an asymmetry of the speed memory in the brainstem after peripheral lesions; however, it may also be missing [116]. Rarely, it even occurs in the context of central disorders [117], [118], [119].

A vertical nystagmus after provocation is called perverted head-shaking nystagmus. It is an indicator for a central lesion that is observed for example in cerebellar disorders [120].

When fixing a stationary target in straightforward position, it is possible to examine if a spontaneous nystagmus as sign of a peripheral lesion can be suppressed or (in cases of central disorders) rather increases [59]. Recent studies, however, could show that insufficient fixation suppression is a rather unspecific hint for cerebellar infarction, which is only observed when the nodulus is concerned [121]. 

Gaze-evoked nystagmus can be classified topographically-anatomically based on its type. For completion, the orienting examination of smooth pursuit movements, the saccade system and the optokinetics (optokinetic drum) is recommended [114], [115].

Frenzel goggles allow evaluating a spontaneous nystagmus during straightforward gaze that possibly evades visual resolution. Frenzel goggles can also be used after head-shaking nystagmus.

The cover test that is not widely distributed in otolaryngology is an important procedure to differentiate central disorders. It is helpful to detect misalignments of the eye axis. This test is of crucial importance in the context of identifying an acute vestibular syndrome [94], [114], [115].

The clinical head impulse test (HIT) is a modern and meanwhile essential part of orienting examination in the context of vertigo. For the clinical (bedside) head impulse test, the examiner looks into the patient’s eyes. The patient is asked to fix for example the nose or the middle of the forehead of the examiner. Then about 5–10 head impulses are performed in the level of the horizontal semicircular canals, preferably from the middle position in lateral direction. For performing the head impulse test, it must be considered that a sufficiently high speed of the head is necessary (>150°/s). If the speed is too low (about <100°/s), the smooth pursuit system may still be active. If the speed is too high (>300°/s), the stimulation might approach zero [38], [39]. There should be enough light in the room, the distance between the examiner and the patient should be about an arm’s length. Ocular particularities and irregularities of vision should be taken into account and possible problems with the cervical spine have to be mentioned. It is recommended to perform the head impulses in an irregular and non-predictive way regarding the side. The patient should remain passive so that the movement of the head cannot be anticipated, which might influence the result [38], [39].

**Conclusion:** Orienting examinations such as the analysis of standing, walking, disorders of eye movement, nystagmus, and the oculomotor aspects are necessary for plausibility checks and provide qualitative findings. 

### 6.2 Clinical examination for acute vertigo

A standardized procedure for acute vertigo could only be established in the last years [46], [47], [48], [49]. In the context of anamnestic and orienting diagnostic hints for acute symptoms of vertigo (acute vestibular syndrome) and criteria of unilateral vestibulopathy (vestibular neuritis) such as rotational vertigo, horizontal-rotating spontaneous nystagmus, tendency to fall, gait disorders, nausea, and vomiting, the differentiation between unilateral peripheral vestibulopathy and central disorder is in the focus. The physician has to face the difficulty that an acute unilateral vestibulopathy (vestibular neuritis) may show exactly the same symptoms as stroke (pseudoneuritis), e.g. as consequence of a brainstem or cerebellar infarction. Acute unilateral vestibulopathy is interpreted as emergency, rapid treatment and an interdisciplinary approach are required.

The examination sequence for differentiation can be performed without any technical system in the emergency room (bedside). It is of highest priority before other diagnostic procedures and may lead to immediate therapeutic consequences (e.g. intravenous thrombolysis, time frame of about 4.5 hours) [122].

Cnyrim et al. [46] were the first to confirm that it is possible to differentiate a peripheral vestibular cause from pseudoneuritis by applying a combination of different orienting test procedures. In this context, the examination of the oculomotor findings by means of the cover test (skew deviation) is crucial since it is considered as very specific test procedure.

Newman-Toker et al. [47] and Kattah et al. [48] could show that the clinical head impulse test has a crucial significance for the detection of central disorders (e.g. AICA, PICA infarction). In this study, the majority of the patients with stroke had a negative head impulse test (hVOR) (>90%). Only in some cases, a positive head impulse test was found despite central lesions.

Currently, there are 2 possible procedures that can be performed at the bedside without the need of technical devices. Both procedures are highly sensitive and highly specific and in the acute stage superior to imaging (MRI) [123], [124].

#### 6.2.1 5 steps procedure (“the big five”)

The 5 steps procedure (“the big five”) [46], [49] is an algorithm for orienting examination of acute vestibular syndrome. The following steps are recommended:

- **Cover test:** If skew deviation is found, a central lesion is probable.
- **Differentiation between a peripheral spontaneous nystagmus and a central fixation nystagmus by means of fixation suppression and Frenzel goggles:** If the spontaneous nystagmus can be suppressed and if it increases with fixation, a central lesion is probable.
- **Examination of the eyes in the main gaze positions:** If a gaze-evoked nystagmus in the opposite direction of the spontaneous nystagmus is diagnosed, a central disorder is probable.
- **Examination of the slow smooth pursuit:** If a saccade of the smooth pursuit is diagnosed, a central disorder is probable.
- **Performance of the clinical head impulse test for hVOR:** If catch-up saccades are missing, a central lesion is probable.

#### 6.2.2 HINTS test

The method described by Kattah et al. in 2009 and Newman-Toker (**h**ead **i**mpulse **n**ystagmus **t**est of **s**kew) includes the application of 3 clinical, non-device-based tests [47], [48]:

1. **Clinical head impulse test for hVOR:** A normal result indicates a possible central lesion.
2. **Examination of the eyes in the main gaze positions:** The presence of a spontaneous nystagmus and a gaze-evoked nystagmus in the opposite direction of the spontaneous nystagmus is a possible indicator for a central lesion.
3. **Cover test:** If a skew deviation is found, a central lesion is probable.

Only in rare cases (<10%) with findings indicating a possible peripheral lesion, also a central vestibular lesion may be present. Acute vertigo without nystagmus is rare but it has been described in the acute phase of cerebellar infarction [125]. Acute, combined peripheral and central disorders have been published as single case descriptions [[126]. In single cases, even cerebral infarction may be present with a positive head impulse test [127], [128].

All this reveals that the differentiation between peripheral and central causes is still difficult. So it is important to collect further aspects that indicate or exclude central lesions. Accompanying cranial nerve lesions and headaches are indicating symptoms. But also concomitant hearing disorders may indicate a central cause [128], [129], [130].

Technical examination methods in the field of otolaryngology that also include hearing tests increase the diagnostic evidence and are appropriate for quantification of disorders. The result of the orienting examinations then leads to the treatment concept.

Despite clear progress in the diagnostics of vertigo, only few evidence-based evaluations for orienting diagnostic procedures are available [131].

**Conclusion:** In the context of acute symptoms with the leading symptom of vertigo (acute vestibular syndrome) currently orienting tests are helpful (e.g. five-step procedure [46], [49] and HINTS test [47], [48]), in order to differentiate acute unilateral peripheral causes from a central genesis with high diagnostic probability. Hereby, the clinical head impulse test and the cover test play a key role.

## 7 Modern technical examination of the vestibular system

In the last years, modern technical vestibular diagnostics, in particular the video head impulse test and VEMP diagnostics, led to the fact that a complex examination of the vestibular reflex structures can be realized. Those examination methods are increasingly wide-spread in Germany [37], [38], [39], [42], [43], [44], [45]. Also the application in the context of ENT-specific expert opinions is more and more established [56].

While orienting examination methods give a qualitative statement about vestibular disorders, the severity of the impairment can be quantified by means of technical measurements. The possibilities of technical diagnostics are manifold (Table 3 (Tab. 3)). In the following, modern procedures, video head impulse test, and the diagnostics by means of cervical and ocular vestibular-evoked myogenic potentials are intensively described and their importance and benefit for clinical practice are critically discussed.

### 7.1 Diagnostic procedures of VOR with the video head impulse test

In contrast to the qualitative clinical head impulse test, the video head impulse test measures and visualizes the VOR. The relationship between head and eye movements can be separately displayed for each of the 3 semicircular canals of both sides in the time course. The video head impulse test is currently the only method for examination of the vertical semicircular canals. The method is best evaluated for the analysis of the horizontal VOR (hVOR). The findings can be documented and stored as video file. It is an objective analysis. From a physiological point of view, highly-frequent parts of the VOR (about 3–5 Hz) are measured while the caloric test reflects the low-frequency range of the VOR (about 0.005 Hz) [37], [38], [39], [132], [133].

In clinical routine, the video head impulse test is increasingly applied. Experiences made up to now have shown that its main application area is the detection of peripheral vestibulopathies. Because of the key role of the clinical head impulse test in the differential diagnosis of the acute vestibular syndrome, the vHIT, which is superior to the clinical head impulse test, is essential in emergency units. According to McDougall et al., the vHIT provides results that are similar to the results of the scleral search coil method [134], which is the current standard procedure of 3-dimensional analysis of eye movements (e.g. [14], [18], [19]). The vHIT is a non-invasive procedure and independently from the condition of the external and middle ear, it can be performed with relatively low durations (about 10 minutes) [135]. The diagnostics by means of vHIT is also possible in pediatric patients [136].

#### 7.1.1 Anatomical and physiological basics

The adequate stimulation of all receptors of the semicircular canals is the angular acceleration stimulus, which explains the term of angular VOR. The system of the semicircular canals is present in pairs and assesses rotational accelerations in all 3 spatial dimensions. Head acceleration stimuli are forwarded via the receptor and the subsequent neurons within a very short time, nearly without any delay (about 7–10 ms), to the eye muscles. In daily life, this occurs without being noticed. Vestibulo-ocular reflexes (VOR) count among the most rapid reflexes of the human body. Together with the above-mentioned systems of eye movement and under healthy visual conditions, they realize e.g. a stable image on the retina with all daily movements (gaze stabilization). The VOR is a central part of the sense of orientation [38], [39], [132].

The vHIT examines the ability of gaze stabilization. In the 3-dimensional space, all 3 semicircular canals can be stimulated side-specifically in the according optimal working level. The horizontal semicircular canals are stimulated selectively with a head movement in the horizontal plane (HOR). The vertical semicircular canals that form an angle of about 90° (top view on the head) are arranged in that way that the left anterior and the right posterior semicircular canal are located in one plane. These optimal stimulation planes are defined according to the position of the semicircular canals as RALP (right anterior left posterior) and LARP (left anterior right posterior) planes. For the video head impulse test, the “measurement” of the VOR is performed in the same way as for the clinical head impulse test with head accelerations in all planes of the semicircular canals (HOR, RALP, LARP). The particular arrangement of the semicircular canal pairs causes an antagonistic response (push-pull principle), which ensures a simultaneous inhibition (“push”) and excitation (“pull”) of vestibular neurons. The stimulation of each semicircular canal receptor by head impulses leads to a selectively measurable reflective eye movement (VOR) for each of the 3 semicircular canals [38], [39].

#### 7.1.2 Performing diagnostics and measurement conditions

From a technical point of view, the video head impulse test consists of very light video goggles with an integrated gyroscope for measuring the head movement including soft- and hardware (e.g. laptop). During examination, the examiner stands behind the sitting patient. The patient fixes a stationary target at eye level with eyes wide open (distance about 1.5 m). The light in the room should be sufficient. Before each measurement, calibration should be performed. Contact lenses may be used. In the literature, different methods that are of equal value from a clinical point of view are described for the stimulation (e.g. grasping the head, head movement along the horizontal mandibular branch, movement from the middle to the outside or “from the outside to the inside”) [137], [138], [139].

About 5–10 head impulses should be performed to each side in all optimal stimulation planes (horizontal: HOR, right anterior-left posterior: RALP, left anterior-right posterior: LARP). The angular velocity induced by the head impulses should amount to about >150°/s, the rotation of the head should not exceed 20°. The induced VOR in the time course is influenced by the dimension of the head rotation and the head movement. In cases of intact VOR, the head and eye movement is finished after about 100–200 ms. During the examination, the induced head impulses and the sequence of the eye movement can be observed and demonstrated in real-time via a monitor [38], [39].

#### 7.1.3 Assessment of the results

For evaluating the results, currently the gain of both sides, the gain asymmetry [%], and the occurrence of correction saccades are important factors. In this regard, the hVOR is best investigated up to now. For diagnostics of the vertical semicircular canals, there is currently only little experience [35], [36], [140], [141].

Depending on the device type and the examination conditions, the gain value (relationship between the movements of the eyes and the head) of both sides amounts to a bit less than 1 in healthy persons (about 0.8–1). If the eye movement is reduced, also the gain decreases. The systems available on the market apply different algorithms to measure the eye movement as well as different mathematical methods to calculate the gain value. Alhabib and Saliba identified 6 systems worldwide to perform examinations in practice. The limit value for a normal hVOR gain was very stable with different systems (<0.79 to <0.81) [142].

The side ratio of both gain values is called gain asymmetry given in %. A gain side ratio of >8.5% is considered as pathological [143].

Correction saccades are reflective saccades that manifest when the VOR is disturbed. An impairment of the VOR leads to the fact that the eye can no longer follow the head movement in case of impulse-like stimulation and thus cannot achieve gaze fixation. The eye is delayed in relation to the head movement. In order to keep a stable fixation, it must perform correction in the sense of a saccade (refixation saccade). If the VOR is disturbed, correction saccades are induced in form of covert (i.e. hidden in the cycle of head-eye movement) and overt saccades (i.e. after the end of the head impulse) [35], [36], [143], [144]. In the context of chronic peripheral vestibulopathies, only 3 variants of correction saccades could be observed: isolated covert saccades, uniquely overt saccades, and a combination of both saccade types. There was no correlation between the saccade type and the disease. In our opinion, saccades with low amplitudes (about <50°/s) have no pathological significance [143]. Mossman et al. found out that the gain value of the hVOR is reduced by 5–10% in higher ages [145]. Compensatory saccades did not show any dependence from the age. Other teams could also reveal a reduction of the gain value in higher ages (>70 years) [146], [147], [148], [149]. Anson et al. indicated a correlation of the gain reduction with increased amplitudes of correction saccades in higher ages. They explain this observation with an impairment of highly-frequent, irregular vestibular type 1 fibers by the aging process [146], [147]. Hence, the interpretation of the findings in elderly patients (>70 years) has to be performed with respectively corrected reference ranges. Correction saccades without suspected pathology may appear more frequently in higher ages. The exact identification of physiologically or pathologically overt saccades, however, is difficult in older patients [146], [147], [148].

An acute unilateral, vestibular disorder mainly influences the gain on the side of the lesion. But also the contralateral side should be examined. In the context of unilateral vestibulopathies, for example a reduction of the hVOR gain may occur on the healthy side [150]. This gain change is observed for example in cases of vestibular neuritis in the sense of vestibular compensation. Assessing the gain values and the gain side ratio according to the reference ranges in those patients, the image of bilateral vestibulopathy may present with reduced gain values on both sides. The origin of these findings is not fully understood at present. Possible causes such as central regulatory processes but also modulations of the efferent nerves are discussed [150], [151], [152], [153], [154]. Also previous damage of the VOR gain may lead to difficulties in evaluating the results of the vHIT.

**Conclusion:** The video head impulse test objectively reflects the highly frequent function of the VOR. It is the only technical method for testing the vertical semicircular canals. For the assessment, overt and covert correction saccades, the ratio of head and eye movement (gain value), and the gain value of both sides (gain asymmetry) are relevant.

#### 7.1.4 Clinical vs. video head impulse test

The clinical head impulse test is expected to be able to provide results of the hVOR test with high test efficiency that speaks for or against the objective presence of a vestibular disorder. Current investigations, however, could show that the sensitivity of those tests amounts to only 66% (specificity of 86%). The positive predictive value amounted to 44% and the negative predictive value to 96% [154]. Beside subjective estimations (e.g. examiner’s experience) and different test methods (e.g. head velocity and rotation), also the type of correction saccades (overt or covert or the combination of both) and the impairment of the hVOR gain play a key role [154].

Covert saccades cannot be detected by visual analysis. As already explained, they are hidden in the cycle of head-eye movement. It is also possible that early occurring overt saccades are not identified with the clinical HIT.

How often do covert saccades appear in the acute and chronic stage of vestibular disorders? For the acute vestibular syndrome, no investigations are available. Regarding different chronic peripheral vestibulopathies, a value of about 15% could be calculated [143]. Peripheral vestibulopathies may present with isolated covert saccades that the examiner cannot recognize by means of the classic test procedure of the clinical head impulse test. Tjernström and Magnusson discovered that the head deflections with the higher amplitude lead to a unmasking the covert saccade (uncover test). This “trick” may be applied in clinical practice when performing the clinical head impulse test [155].

Higher-grade impairment of the hVOR or a more important side difference of the caloric test lead to a higher sensitivity in cases of unilateral vestibulopathies with clinical head impulse test [40], [41]. This statement for the clinical HIT that is important for the practice was recently confirmed by vHIT examinations [154], [156], [157]. As limit, a side difference of the caloric test of about 40–50% is considered. So, in cases of higher-grade impairment of the hVOR, also the sensitivity of the clinical head impulse test increases significantly [154].

The neural damage pattern in unilateral vestibulopathies seems to play a crucial role. Damages of quick vestibular type 1 fibers seem to play a subordinate role for the development of pathological caloric tests or significant impairment of the quick nerve fibers had to happen until also the head impulse test shows pathological results. This observation also reveals that the video head impulse test and the caloric tests will not provide identical findings. 

**Conclusion:** The clinical head impulse test mainly provided qualitative results (occurrence of an overt correction saccade) of the hVOR (e.g. horizontal semicircular canals). With the uncover test [155], covert saccades may be unmasked. The video head impulse test has a higher diagnostic precision than the clinical head impulse test.

#### 7.1.5 Video head impulse test and caloric irrigation

With the introduction of the video head impulse test, the question had to be asked if it might be the only test procedure in vestibular diagnostics that can replace caloric irrigation. Prematurely, the statement was issued that there was no longer any need to perform caloric testing, which was the most important method up to then [158].

Regarding the significance of caloric tests and vHIT in vestibular diagnostics, meanwhile confirmed knowledge is available about testing the hVOR:

The caloric test mainly assesses the function of the lateral semicircular canals [159]. In particular, the hVOR is tested. The occurrence of the VOR response is probably based on several factors; however, gravity is not the only one [160]. Caloric and neural effects as well as the central velocity storage modulate the VOR response [161]. The video head impulse test is based on a direct analysis of the VOR checked during gaze stabilization in the context of relatively high-frequent stimulation due to irritation of quick nerve fibers of the VOR [162], [163], [164], [165].

Probably other mechanisms are responsible for the occurrence of the VOR response as consequence of caloric testing compared to the video head impulse test [160]. Higher-frequent responses that are preferably mediated by irregular type 1 afferent nerve pathways play a crucial role for the high-frequent video head impulse test. Regarding the low-frequent caloric test, apparently other, slowly firing vestibular neurons and the central velocity storage are involved [162], [163], [164], [165]. A selective “damage” of the quick type 1 fibers would then lead to conspicuities of the video head impulse test (gain reduction on the affected side, occurrence of refixation saccades). An impairment of the nerve fibers that are not involved in the results of the video head impulse test would be reflected in a pathology detected by the caloric test (side difference of >25%).

When both methods are applied for defined vertigo syndromes, it becomes obvious that the results may be actually different. In practice, cases with normal caloric test results and pathological video head impulse test, cases with normal result of the video head impulse test and pathological caloric tests as well as mixed types with pathological results in both tests are observed [157], [166].

Current investigations of peripheral vestibulopathies could reveal the following particularities: In patients with a vestibular schwannoma, the caloric test has a higher sensitivity than the vHIT [36], [167]. The side asymmetry correlates with the tumor stage [167]. Because of the preferred affection of nerve fibers of the low-frequency range represented by the vHIT, the caloric test is essential for the diagnosis of vestibular schwannomas. Independent from the tumor size, patients suffering from chronic vertigo because of vestibular schwannoma, are identified earlier and more reliably as sick by means of caloric testing than with the video head impulse test (hVOR) [161].

In cases of Menière’s disease, pathological test results of the video head impulse test are also found more rarely [168], [169]. In the majority of patients with vestibular migraine, the caloric test is more often pathological than the video head impulse test [168].

The vestibular neuritis is often associated with an impairment of the high-frequency range of the hVOR. In the acute stage, a comparison of both methods shows a pathological video head impulse test in about 2/3 of the cases. In the time course (including those patients who experienced partial or complete recovery), the ratio was changed so that the video head impulse test was pathological in about 1/3 of the cases [157], [163], [164], [165], [166].

Singular observations exist regarding the large vestibular aqueduct syndrome (pathological caloric test, negative vHIT) [170].

So there seems to be a disease-specific image of the impairment in the context of peripheral vestibulopathies when the hVOR is tested by means of caloric test and vHIT. The results of the caloric test and the video head impulse test are often dissociated. This may also be explained by a damage of different fiber parts (see above) of the VOR. 

**For the clinical practice, currently the following consequences result:**

- A. In cases of acute disorders (acute vestibular syndrome) the application of the video head impulse test has priority before the caloric test because of its higher sensitivity and specificity.
- B. According to the current data situation, in most peripheral vestibulopathies with chronic affection an increased percentage of conspicuities in the caloric test is observed compared to vHIT. If the disease is not known, the caloric test should be performed before vHIT.
- C. vHIT and caloric test are essential in the technical vestibular diagnostics and do not compete with each other; they rather allow an enlarged frequency-dynamic assessment of the hVOR. For differentiated diagnostics, both methods must be applied.
- D. As of a side asymmetry of about 40% in the caloric test, a positive HIT or vHIT can be expected. A positive vHIT most often also indicates a pathological caloric test. From a pragmatic point of view, caloric tests are not necessary in such cases. Some authors believe that it is appropriate to start the diagnostics with the video head impulse test [166], [171]. According to current knowledge, it is reasonable in cases of suspected vestibular neuritis.

The frequency-specific particularities of the hVOR led to the concept of a differentiated analysis of the vestibular receptor functions [37]. It includes among others an extensive analysis of the hVOR by means of further test procedures. This statement is implemented in the overall estimation regarding the status of the 5 receptors and the subsequent reflex pathways [37].

**Conclusion:** Video head impulse test (high-frequency test procedure) and caloric irrigation (low-frequency test procedure) reflect different functions of the VOR. In the context of peripheral vestibulopathies, the image of the disorder is disease specific and not consistent when both test procedures are applied. When a side difference of about >40% is found in the caloric test procedure, the video head impulse test (hVOR) mostly reveals pathological results. 

#### 7.1.6 Video head impulse test in the emergency department

The comparability of studies that have evaluated the leading symptom of vertigo and the incidence of stroke is rather limited for example with regard to the inclusion criteria. According to the current study situation, it must be expected in an emergency case with vertigo that a central neurological complication is found in about 2.5% of the cases [171], [172], [173], [174], [175], [176].

Recently, Mantokoudis et al. [177] have established diagnostic criteria based on the video head impulse test and the caloric test that might indicate stroke or peripheral vestibulopathy as emergency (acute vestibular syndrome). A high grain difference in the vHIT speaks for vestibular neuritis. In cases of stroke with involvement of the PICA, a hVOR gain value is normal in most of the patients. However, the caloric tests show pathological results in up to 22% of the cases. A lower gain difference (<20% gain asymmetry) indicates an AICA infarction. Because of his specific competence, the otolaryngologist is involved in the diagnostics of stroke and thus has a high responsibility in emergency situations. With the use of the video head impulse test combined with orienting examinations, the diagnostic certainty is increased regarding the detection of central neurological disorders.

The clinical evaluation of the video head impulse test in clinical routine is only just beginning. Nonetheless, within a very short time, the test could prevail in the vestibular diagnostic process. Current studies could show that e.g. even alcohol and drugs may influence the results [178], [179], [180]. Furthermore, it became obvious that the highly-frequent hVOR is early impaired by gentamicin. This fact is relevant for the identification of ototoxic disorders (monitoring in cases of systemic gentamicin administration) and therapy control (intratympanic gentamicin application for Menière’s disease) [50], [181].

Recently, detailed analysis of saccades led to new knowledge, but for the application in clinical practice too few investigations are currently available [182], [183]. 

### 7.2 Otolith function: vestibular evoked myogenic potentials

In the last years, vestibular evoked myogenic potentials have become an essential diagnostic instrument for the assessment of vertigo. Hereby, the vestibular stimulation is performed via supra-threshold acoustic stimuli, preferably in air conduction. This method is well evaluated. 

#### 7.2.1 Anatomical and physiological basics

The paired otolith organs have an oval shape, measure about 1–2 mm², and are slightly convex [184]. The utriculus located in the horizontal plane is found nearly in projection on the inferior inner orbita edge; the sacculus in the sagittal plane is found nearly in projection on the lacrimal duct. Due to the inertia of the otoconia found in the otolith membrane in about 2–3 layers, the otolith organs mediate linear acceleration stimuli, head tilting, and the relation to the gravitation vector [185]. The stimulus transmission occurs via the inferior vestibular nerve (sacculus) and the superior vestibular nerve (utriculus). Voit’s nerve is an anastomosis between sacculus and superior vestibular nerve that starts at the hooked part of the sacculus [186]. This double innervation of the sacculus contributes to the fact that some neural impulses reach the superior vestibular nerve via the sacculus when the utriculus is stimulated. In the context of phylogenetic development, acoustically sensitive cells were conserved in both otolith organs. The physiological function of those cells has been lost because it was not subject to evolutionary pressure [187]. The rudimentary acoustic sensitivity of parastriolar vestibular cells in the utriculus and sacculus is used in the context of VEMP diagnostics. With supra-threshold acoustic stimuli (e.g. 100 dB nHL), optimally at 500 Hz, reflective nerve impulses of mainly irregular otolith neurons may be activated [188], [189]. The generated electrical potentials can be objectified by means of surface electromyography of cervical (sternocleidomastoid muscle) and ocular measurements (oblique and inferior rectus muscles) resulting in the typical VEMP curves [23], [24], [25], [33], [34], [44], [45]. Their morphology (amplitude and latency) in the time course and side comparison (amplitude ratio, latency ratio) is used as measure for the function. Regarding the origin of the ocular VEMP measurements, controversial discussions were conducted during the last years, especially with regard to the double innervation of the sacculus [76], [77], [78], [79]. Nowadays it is accepted that cVEMP and oVEMP in air conduction are an indicator for the preponderant sacculus function (sacculo-collic reflex) or utriculus function (utriculo-ocular reflex) [75]. Both methods are more and more applied in daily clinical practice as well as in the context of ENT specific expert opinion. VEMP can be applied in all ages [44], [45], [56].

#### 7.2.2 Cervical vestibular evoked myogenic potentials

In 1992, Colebatch and Halmagyi were the first to publish a report about the methods of cervical vestibular evoked myogenic potentials [23]. In practice, the measurement via the superior part of the sternocleidomastoid muscle could be established [33], [34], [44], [45].

Prior to each VEMP examination, ear microscopy (if needed removal of earwax, assessment of the condition of the auditory meatus and eardrum) as well as audiological diagnostics (e.g. tympanometry, tone audiometry, measurement of air and bone conduction thresholds) are recommended. According to the severity, conductive hearing loss leads to a reduction of all VEMP responses. Sensory hearing loss (also deafness), however, does not impair the examination of the VEMP.

For daily routine, measurements by means of air conduction are currently best evaluated. Bone conduction stimulation is possible in cases of conductive hearing loss. For examination by air conduction, in-ear headphones or earphones are suitable. Precondition for VEMP measurement is a VEMP module. The surface electrodes can be placed for example in the area of the upper third of both sternocleidomastoid muscles, in the middle of the forehead (neutral electrode), and in the area of the jugulum (reference electrode). For a rapid and undisturbed measurement, low impedances (e.g. <5 kΩ) are necessary. For measurement in sitting or lying position, the head is turned to the contralateral side and slightly inclined or lifted so that the sternocleidomastoid muscle of the stimulated side is contracted. During cVEMP measurements, the simultaneous control of muscle pre-tension by means of EMG was established [190], [191], [192]. According to the current international recommendations [192], first the frequency of the best acoustic sensitivity of the otolith organs is selected as stimulus frequency (500 Hz). At this frequency, the lowest thresholds and the highest amplitudes are found. As stimulus, click or burst stimuli are suitable. Clicks, however, have relatively broad frequency response with high-frequency parts and the frequency-specific stimulation is more unspecific than for burst stimuli [193]. For stimulation, for example 50–100 repetitions are suitable until a typical amplitude complex at e.g. 100 dB (nHL) results. cVEMP are inhibitory reflex responses. During ipsilateral stimulation, as objective sign of an intact sacculo-collic reflex, ipsilateral biphasic muscle potentials are found (positive potential at about 13 ms and negative potential at about 23 ms) as well as acoustically generated potentials at about 33 ms and about 44 ms of which the properties are not exactly evaluated yet [33], [34], [44], [45]. The amplitudes (peak-to-peak measurement) are subject to fluctuations according to the patient’s age and amount to about <500 µV.

#### 7.2.3 Ocular vestibular evoked myogenic potentials

Since the nerve fibers of the utriculo-ocular reflex cross centrally to the contralateral side, the measurement of the muscle potentials for oVEMP is performed in air or bone conduction of the contralateral side. The surface electrodes for analysis of the oVEMP can be placed for example bilaterally at the lower edge of the orbita and the reference electrode parallel below. For the neutral electrode, the middle of the forehead is recommended. Recently, Govender et al. have suggested a modified electrode position that also promises high oVEMP amplitudes [194]. During stimulation, the patient has to look upwards in order to contract the outer eye muscles (oblique and inferior rectus muscles).

oVEMP are excitatory electromyographic responses. An electromyographic control of the muscle tension during the measurement is not necessary. The classical oVEMP potential is also biphasic (negative potential at about 10 ms, positive potential at about 5 ms). The amplitudes are lower than for cVEMP (about <20 µV) and achieve partly the nanovolt range [33], [34], [42], [43], [44], [45]. The angle in upward direction and the horizontal gaze deflection as well as the body position influence the oVEMP results. The head rotation and the vision have no significant impact on the results [195].

#### 7.2.4 Assessment of the results

VEMP measurements in air conduction (500 Hz) are the classical stimulation method in daily practice. For assessment of the results, the amplitude (measurement between the amplitude maxima [µV]) of the received measurement and the respective latency (s) as well as the amplitude ratio (asymmetry ratio = AR [%]). For calculation of the AR, the higher (h) and lower (l) amplitudes (A) of both sides are considered: AR = 100 (Ah-Al)/(Ah-Al). An amplitude ratio of >50% is pathological [192]. Thus quantification of the findings is possible. The data of the reference ranges varies relevantly in the literature [33], [34], [141], [192].

Pathological latencies and amplitude ratios indicate an impairment of the respective reflex. However, the entire reflex chain has to be considered because the otolith organs as well as the superior and inferior vestibular nerves, central pathways, or rarely even muscle functions may be impaired [196]. The application of VEMP requires a previous differential diagnostic classification of the complaints. 

The reduction of the muscle pre-tension (cVEMP) with increasing age [190] and the age-associated rarefication of the vestibular nerve fibers and sensory cells, among others in the utriculus and sacculus [197] contribute to the fact that VEMP measurements at about the age of 60 years and onwards are no longer successful in 100% of the cases [198], [199]. This may make interpretation of the VEMP and thus the evaluation of the otolith function in higher ages more difficult.

Different stimulation modalities (e.g. type of stimulus, location of stimulation, stimulus frequency) have a significant impact on the VEMP amplitudes [33], [34], [44], [45], [200].

**Conclusion:** Cervical and ocular VEMP in air conduction (500 Hz) reflect the main sacculus and utriculus functions. For assessment of the results, the amplitudes, latencies as well as the amplitude ratio are used. Regarding the cVEMP, the muscle pre-tension must be taken into account for evaluation of the reference range.

#### 7.2.5 Modifications of VEMP diagnostics

Beside stimulation by air conduction, in the literature the stimulation via bone conduction is described, for example by means of conventional bone conduction headphones, mini-shakers, or reflex hammers.

Stimulation via bone conduction may be performed for example in the middle of the forehead or in the area of the mastoid [33], [34], [44], [45], [201]. It may be suitable when conductive impairment (e.g. perforation of the eardrum, open mastoid cavity) is present. Govender et al. assumed different mechanisms for stimulation of the otolith organs. Bone conduction stimuli should rather lead to stimulation of the otolith membrane while air conduction stimuli should lead to direct stimulation of acoustically sensitive vestibular hair cells [202].

Beside the optimal stimulus frequency of 500 Hz, the acoustic stimulation of the otolith organs is also possible with other stimulus frequencies (<100 Hz – >4 kHz). A completing VEMP analysis is suitable for example in the context of traumatic disorders and peripheral vestibulopathies with changed inner ear mechanics [193], [203]. Sandhu et al. and Kim-Lee et al. could confirm that Menière’s disease is associated with frequency dynamic changes with shift of the VEMP amplitude maxima to the higher-frequency range (up to 1 kHz) [204], [205]. Frequency dynamic changes were also described for dehiscence syndrome [206], vestibular migraine [207], and in higher ages [199], however, only little evaluated. For VEMP multi-frequency analysis, we recently suggested the simultaneous application of several stimulus frequencies with a special burst stimulus [208]. In this way, several frequencies may be measured at the same time in one examination with control of the applied acoustic energy.

Since VEMP examinations are supra-threshold diagnostic measures, it is recommended to apply the VEMP stimulus in that way that the acoustic energy is kept low [209], [210]. Mattingly et al. recently reported about hearing loss during VEMP diagnostics [211].

The application of chirp stimuli seems to be a promising modification of VEMP analysis, especially in the context of diagnostics of frequency dynamic changes of the otolith organs [193], [212]. Chirps are special stimuli with a frequency range that can be flexibly designed. We could recently show that high VEMP amplitudes can be generated with chirp stimuli that were constructed for narrowband and broadband frequency structures [193], [212]. The clinical experience, however, is rather low up to now.

#### 7.2.6 VEMP in clinical practice

Recently, numerous articles have been published showing that VEMP measurements contribute to a decisive improvement of vestibular diagnostics. For the first time, it is possible to objectively assess the involvement of the otolith organs in vestibular syndromes but also isolated disorders of the otolith function in an easy way [33], [34], [37], [44], [45], [213], [214].

In the context of vestibular neuritis, cVEMP and oVEMP allow statements about an involvement of the otolith organs. If only the cVEMP measurements are pathological, an involvement of the inferior vestibular nerve (or the sacculus function) can be assumed. If additionally oVEMP examination is conspicuous, an involvement of the superior vestibular nerve (or the utriculus function) can be expected. VEMP confirm the observations that have already been made by Fetter and Dichgans [14] regarding VOR diagnostics that different differential diagnoses may lead to vestibular neuritis because of separate neural affections of the superior and inferior vestibular nerves. Neuritis of the inferior vestibular nerve [215] is a newly identified disease with functional disorder of the sacculus (pathological cVEMP) and the posterior semicircular canal. The clinical complaints are moderate. Staggering vertigo is the dominating symptom. Spontaneous nystagmus may be lacking [216].

In the diagnostics of dehiscence of the semicircular canals, VEMP play a key role. Govender et al. found conspicuous cVEMP in 85% of the cases. In 62% of the patients, oVEMP were pathological. A dehiscence syndrome is suspected when reduced VEMP thresholds are found in air conduction stimulation with 500 Hz as well as increased VEMP amplitudes [217]. Hunter et al. described that cVEMP and oVEMP amplitudes and cVEMP thresholds correlate with the severity of a dehiscence [218]. According to Brantberg and Verrecchia, the stimulation with 90 dB nHL is sufficient to confirm the clinical suspicion of dehiscence syndrome [219].

Recently, some groups published an article about pathological oVEMP in the context of benign positional vertigo [220], [221], [222]. Hereby, the correlation of this disease with a disturbed utriculus function [223] can be easily objectified [222], which had to be confirmed formerly by complex rotatory tests.

VEMP examinations are suitable for all peripheral vestibulopathies [33], [34], [44], [45] as well as for vestibular migraine [207] in order to identify involvement of the otoliths. In cases of Menière’s disease, the assessment of several frequencies is useful because an endolymphatic hydrops apparently leads to a changed frequency behavior of vestibular neurons [224], [225], [226].

In the last years, VEMP were also applied for control after labyrinthine interventions (e.g. normalization of the threshold and amplitude after surgery of semicircular canal dehiscence), therapy control of Menière’s disease (after intratympanic gentamicin administration [50], [51], [52]), and in the context of perioperative neuromonitoring [53], [54].

**Conclusion:** VEMP results provide detailed information about the otolith function and thus differential diagnostic information in the context of peripheral vestibulopathies. They may facilitate therapy decisions.

## 8 Other modern vestibular test procedures

### 8.1 Dynamic visual acuity

Since the dynamic visual acuity test (DVA) [227] is based on subjective statements regarding the fixation object during the video head impulse test. However, this requires a normal or corrected visual acuity when fixing the object [228]. Automated variants (e.g. the use of Landolt rings) show a high exactness of the test and allow a differentiation of vestibulopathies [229], [230]. The test variants are still only little distributed in practice.

### 8.2 Vibration-induced nystagmus

In 1973, Lücke was the first to observe that nystagmus can be induced by means of vibratory stimulus (100 Hz) near the mastoid [231]. This test allows diagnosing asymmetries of the VOR in cases of vestibulopathies. Hamann and Schuster revealed that the vibration-induced nystagmus (VIN) goes into the direction of the non-affected side of patients with peripheral vestibulopathies [232]. Koo et al. concluded based on their investigations that the VIN can be compared to caloric tests and leads to better results than orienting examination by means of provocation (head shaking) [233]. Perez et al. stated that the velocity of the slow nystagmus phase is relatively low [234]. Dumas et al. could observe that a caloric nystagmus may be inverted [235]. The VIN complements other test procedures. Its diagnostic precision is estimated very high. The test is side-specific and independent from the condition of the ear. A selective analysis of the semicircular canal and VOR functions is not possible. It is not fully clarified yet which frequency range of the VOR is exactly stimulated. The distribution in the practice is still relatively low.

## 9 Differentiated vestibular functional analysis by means of modern diagnostic procedures

**Figure 1:**
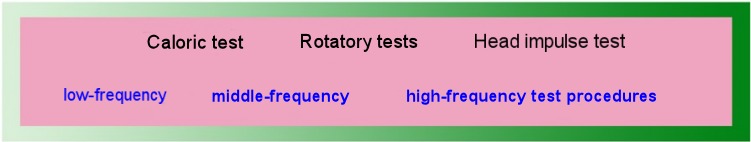
Areas of the hVOR and assessment with diagnostic methods (according to Walther, 2013, taken from [94])

Today, the presented modern test procedures (video head impulse test and VEMP) and technical methods of vestibular diagnostics (e.g. caloric stimulation, subjective visual verticals, rotatory tests, see Table 3 (Tab. 3)) lead to a high diagnostic certainty regarding an exhaustive functional estimation of the 5 receptors of the vestibular organ and the subsequent reflex pathways. Since all 5 sensory elements of the vestibular organ can be assessed, the term of “5-receptor diagnostics” was used in the past [56]. Considering frequency-specific statements of the test procedures (Figure 1 (Fig. 1)) and the possibility of topological analysis of all 5 vestibular receptors, a differentiated side- and sensor-specific assessment of the vestibular function with receptor proportion and subsequent reflex pathway can be performed. The relevant advantage of modern procedures is their objectivity. Beside a topological and frequency-specific statement, a differentiated vestibular functional analysis [37] also considers the possibility of assessing the time course (e.g. evaluation of disease courses with regeneration, partial damage or missing functional recovery, and monitoring after therapeutic interventions or ototoxic therapy). In the context of multi-step analysis procedures, objective, technical test procedures, video head impulse test, VEMP as well as caloric tests have high priority [37].

Due to their comparably easy and time-saving performance in vestibular diagnostics, modern procedures are more and more established in daily practice. Subjective and unspecific as well as cost- and time-intensive procedures are increasingly replaced by specific, objective, quantitative, and economic methods [236]. However, complex diagnostic procedure and established methods do not lose their significance. They significantly complete the procedures and increase the diagnostic precision. With modern methods, vertigo is no longer a diagnostic chameleon but in most of the cases, it can be treated.

**Conclusion:** Modern diagnostics provide objective, side-specific, and receptor-specific quantitative information about the integrity of vestibular reflexes. Beside this topological analysis, frequency-specific statements about the function in the timely course are possible.

## 10 Modern interdisciplinary diagnostics for vertigo and dizziness

Many diseases with the primary or accompanying symptom of vertigo can be diagnosed without interdisciplinary contributions and submitted to appropriate treatments. In some cases of vertigo syndromes, diagnostic differentiation is difficult. Often comorbidities may be present which makes therapeutic decisions rather complex. In the last years, progresses in the interdisciplinary diagnostics have contributed to increasing the diagnostic certainty, which requires a close interdisciplinary cooperation. So interdisciplinary diagnostics are necessary for example in cases of functional vertigo syndromes, functional disorders of the cervical spine with associated vertigo, gait disorders or tendency to fall in higher ages and in the differential diagnostics of vestibular migraine or vestibular paroxysmia. Promising new diagnostic methods compete with current diagnostic standards.

### 10.1 Endolymphatic hydrops

The endolymphatic hydrops is an abnormal dilation of the endolymphatic fluid spaces of the inner ear, it is the pathogenetic correlate of Menière’s disease [90]. In 2007, the group around Nakashima was the first to succeed in 2007 in visualizing an endolymphatic hydrops by magnet resonance imaging after intratympanic application of a diluted gadolinium preparation [237]. This procedure is superior to intravenous enhancing [238] and can be performed bilaterally [239].

In 2016, Ziylan et al. performed an evidence based analysis and compared electrocochleography and intratympanic gadolinium application. In summary, they found – however in few patients – an advantage for intratympanic gadolinium instillation [240]. Side effects with regard to inner ear disorders, especially effects in the hearing ability, cannot be expected according to a recent study. Description and evaluation of the method were meanwhile improved [241]. For the practice, there is a direct medical benefit: diseases that are accompanied by endolymphatic hydrops, in particular Menière’s disease, can be objectively diagnosed for the first time with a higher diagnostic evidence, which has direct therapeutic consequences. This promising method competes with the current diagnostic standards of hydrops identification. Close cooperation between otolaryngologists and radiologists is required.

### 10.2 Functional vertigo syndromes

The most frequent vertigo syndromes observed in practice are those that cannot be fully explained by organic causes or that develop as a consequence of a vestibular disease. Brandt et al. recognized the correlation between mental factors and vertigo syndromes (“phobic paroxysmal vertigo”) [11], [12]. Eckardt-Henn et al. [61] and Dieterich [242] used the term of primary and secondary somatoform vertigo. Both terms are relevant for the daily work of an otolaryngologist. In the context of primary somatoform vertigo, there is no correlate for an organic disease. The secondary somatoform vertigo develops as a consequence of an organic (vestibular) disorder and affects a high percentage of patients with chronic episodic complaints [42], [61]. The early detection is the basis for an early introduction of psychotherapy and thus for therapeutic success [243]. In the last years, Staab et al. developed conceptions that are summarized under the headline of “chronic subjective vertigo” [62], [63], [64]. Currently also the term of “functional vertigo” is used. According to Strupp et al. [2], it occurs in 2 subtypes as “persisting subjective staggering vertigo” and as “phobic staggering vertigo”. A consensus in this context has not yet been found. A classification is being prepared [2] and so, overlapping of the terms in practice is frequently observed.

In cases of recurrent (episodic) vertigo syndromes, especially peripheral vestibulopathies such as Menière’s disease, benign positional vertigo as well as acute, unilateral, incompletely compensated vestibular disorders (“vestibular neuritis”), but also vestibular migraine, often comorbidities are observed. This is in particular relevant for anxiety disorders and depressions [98], [100]. Eckardt-Henn et al. [244] found psychiatric comorbidities in 65% of vestibular migraine and in 57% of Menière’s disease patients. Otolaryngologists ought to know and recognize those correlations. In single cases, this may lead to difficulties, especially when no identifiable deficits are present in the initial stage of the disease that would confirm an organic disorder. Hints may be taken from the anamnesis; an interdisciplinary cooperation with neurologists, psychiatrists, and psychologists is of high importance for diagnosis and therapy.

### 10.3 Vestibular migraine

Vestibular migraine belongs to the most frequent diseases with episodic vertigo. There may be differential diagnostic overlapping with other vertigo syndromes with episodic vertigo (Table 2 (Tab. 2)).

The current classifications of the International Headache Society (ICHD-3 beta version) [245] and the one of the Bárány Society for Neuro-Otology [246], [247], [248] from 2013 are used to categorize the symptoms. According to those classifications, an attack duration of at least 5 minutes is required (Menière’s disease: >20 minutes). Isolated vertigo episodes without accompanying symptoms are possible. Headaches, ear symptoms (e.g. ear pressure, hearing loss), visual aura occur in at least 50% of the cases.

Diagnostic differentiation may be difficult when apart from subjective symptoms no objective impairment can be “measured”. The main differential diagnosis is Menière’s disease.

Disorders of the vestibular functions are rarely found in the acute stage so that the diagnosis must mostly be based on clinical findings. Despite subjective problems, hearing loss is found only in about 38% of the patients [249]. An impairment of the caloric irritability was observed in 22% of the cases, only in 9% of the patients the video head impulse test was pathological [168]. A benign positional vertigo is often observed as associated symptom [249]. Also vestibular migraine can occur as comorbidity of Menière’s disease [249].

When the differential diagnostic classification is problematic, a therapeutic attempt (diagnosis ex juvantibus) may be initiated with beta blockers (on low evidence level).

Cooperation with neurologists is essential in terms of differential diagnostics. The consultative involvement of psychologists and psychiatrists is recommended.

### 10.4 Vestibular paroxysmia

In cases of short-term vertigo episodes that may occur more than 30 times per day, a neurovascular compression syndrome is possible [9], [249], [250]. The causes are arterial or also venous mechanical irritations of the vestibular nerve in the area of the cerebellopontine angle [9], [249], [250]. Other accompanying symptoms may be temporary or permanent hearing loss and tinnitus. So there is differential diagnostic overlapping with Menière’s disease and vestibular migraine. Typical constellations of the findings that may indicate a certain diagnosis are nearly not found in vestibular diagnostics. An international consensus is currently being prepared. The certainty of the diagnosis is also higher when carbamzepin leads to improvement [9], [250].

Magnetic resonance imaging for differential diagnostics also of central processes is the most important interdisciplinary examination performed together with radiologists. Today, the focus is on 3D reconstructions of the cerebellopontine angle. However, also in healthy subjects, conspicuous findings may occur [92], [250], [251].

### 10.5 Falls and unsteady gait in higher ages

Because of the demographic development, falls become an increasingly relevant problem in higher ages [5], [101], [102]. Disorders of the gait are frequent accompanying symptoms. They are often perceived as vertigo or balance disorders. Thus, the identification of their origin is crucial for the otolaryngologist in the interdisciplinary context. 

For the anamnestic assessment of the individual risk to fall, the evaluation of risk factors is recommended [5]. If there are more than 3 risk factors, the risk to fall is increased. A patient who had already fallen has a multiplied risk of falling again. The symptoms of vertigo and gait disorders are associated with an increased risk of falls [5].

Recently, Jahn et al. summarized the most important anamnestic and diagnostic hints for gait disorders in higher ages [73]. The modern diagnostic elements also encompass the analysis of the interrelation between gait and cognition (dual task). In practice, the test with cognitive items (e.g. counting) and at the same time movement is more and more integrated in the clinical examination procedures [73]. Slower walking or even standing are hints for a disturbed cognitive performance (e.g. due to central vascular disorders or dementia). 

For the modern analysis of gait, video systems and pressure sensitive floor mats are used today [73], [101], [102]. Functional methods of magnetic resonance imaging and nuclear medicine are helpful regarding the diagnosis [73]. 

### 10.6 “Cervicogenic vertigo” (disorder of the head-body position)

The term of cervicogenic vertigo is controversially discussed [2], [69], [70], [252], [253], but it is no longer generally denied [68].

However, none of the diagnostic procedures may guarantee a high diagnostic certainty. So it is recommended to search for symptoms that beside the anamnesis include or exclude cervicogenic vertigo or better a disorder of the head-body position. In a recently published case report, Brandt and Huppert described that vertigo appeared in the relation to neck pains after head movements for some days [68]. The authors discuss that a disturbed orientation (vertigo) may develop based on central mechanisms (e.g. misinterpretation of expected or currently present visual somatic and vestibular afferent and efferent signals, “neural mismatch concept”). Based on the results of their investigation, Hölzl et al. assume that a cervico-ocular reflex induced by neck rotation is caused by an upbeat nystagmus in the neck torsion test [254]. 

Beside the examination of the eye movements, targeted functional examination of the skull, the mobility of the cervical spine, the head, neck, and masticatory muscles as well as the mandibular joints is required in cases of clinical suspicion. According to L’Heureux-Lebeau, pains in the area of the spinous processes, sensation of numbness, movement disorders of the cervical joints and a positive neck torsion test with nystagmus allow for the diagnosis of cervicogenic vertigo [255].

Diagnostics require the cooperation with dentists, orthodontists, physiotherapists, and specialists in the fields of osteopathy and manual therapy.

**Conclusion:** Diagnostic assessment of vertigo is only possible based on close interdisciplinary cooperation.

## 11 Modern diagnostics of vertigo in ENT-specific reporting

Specific reporting is a particularity in the context of medical routine. In most of the cases, there is no physician-patient relationship in the classical sense. The criteria are defined by the requesting institution and the legal conditions. The current state-of-the-art is the general basis for the evaluation. Often, the diagnostic findings have to be estimated on a probability scale ranking from simple to utmost probability and depend on the legal conditions [256].

Apart from exceptions (benign positional vertigo [257], Menière’s disease [258]), vertigo is assessed based on standardized guidelines according to Stoll’s tables [259], [260], [261]. The references for the evaluation of vertigo, however, are exclusively subjective statements. Data from the patient’s history (intensity) are related to the requirements of daily and professional life (stress). The ability to cope with pressure results from the correlation of the subjective data (0 = nearly free of complaints up to 4 = severe vertigo and loss of orientation) with attributes like “daily”, “preventable”, and “extraordinary”. Examples of subjective test procedures (Romberg’s test, Unterberger test, tandem Romberg’s test, balancing) are mentioned [262].

Despite this dependence from subjective findings, a differentiated assessment of vertigo is requested based on clinical examination [260], [262]. It contributes to clarifying if and to what extent of a disturbed sense of orientation is plausible. It is also important to know if the main symptoms of the suspected disorder are part of the field of otolaryngology or if additional specific reports are necessary.

History taking with consideration of the degree of subjective impairment at rest and with movement is of crucial importance. Sometimes, professional impairment has to be evaluated. Also for specific reporting, only those procedures may be applied in the context of diagnostics that correspond to the state-of-the-art of the discipline (predominant approval, confirmed evidence and benefit of the method). Reference ranges should always be mentioned. They are the measure for the assessment. Regarding the evaluation of the findings, also ENT specific reporting has to take into account that for example alcohol and drugs may influence the result of modern diagnostics [178], [179], [180].

We recently stated that modern diagnostic procedures such as the clinical and video head impulse test as well as the cervical and ocular vestibular evoked myogenic potentials are currently an essential (“up-to-date”) part of the examination procedure in the context of ENT-specific reporting for vertigo [56]. 

One of the tasks of an otolaryngologist is also the descriptive estimation if vertigo is accompanied by central vestibulopathies or psychic comorbidities. Those may then require additional specific assessment and reporting when significant effects are suspected for the overall assessment.

How can chronic complaints be evaluated after acute unilateral vestibulopathy? Is it possible to correlate them with the results of objective diagnostics?

Patel et al. could show that modern diagnostics (video head impulse test of all 3 canals on both sides) do not correlate with the subjectively evaluated impairment in the context of chronic vertigo complaints. Patients with mild and those with severe complaints had identical findings [263].

McCaslin et al. examined a group of patients with unilateral impairment of the sacculus function (pathological cVEMP) and unilateral vestibulopathy (pathological caloric test, sometimes pathological cVEMP). Patients with an impaired sacculus function had also a significantly impaired postural stability that was nonetheless better than the one of patients with a pathological caloric test alone or additionally impaired sacculus function. Subjectively (assessment by means of DHI), however, no difference could be found [264].

Piker et al. found out that pathological test results for chronic vertigo are much better correlated with psychic symptoms such as anxiety and depression [265]. Since chronic vertigo is associated with psychic comorbidities to a high percentage [98], [100], [244], it should be assessed before overall evaluation if additional psychological or psychiatric reporting is necessary to clarify to what extent otolaryngological aspects are involved in the problem or if comorbidities have a significant impact. 

Findings of modern diagnostics are essential in otolaryngology-specific reporting due to their objective properties. They contribute crucially to the clarification of the question if and to what extent (causal or final) a disorder belongs to the discipline of otolaryngology or not. The currently available literature reveals that it is actually not possible to “measure” subjective impairment by vertigo with objective findings of vestibular diagnostics.

**Conclusion:** The ENT-specific assessment of permanent vertigo is currently performed independent from legal aspects based on standardized tables. A disease-specific assessment is currently only performed in the context of episodic vertigo syndromes.

In cases of otolaryngological reporting, subjective symptoms dominate and thus the subjective evaluation. Modern procedures (video head impulse test, VEMP) are an important element because of their high objectivity. In this way it is possible to clarify if an objective disorder of the vestibular function belongs to the discipline of otolaryngology. Findings of vestibular tests do not correlate with subjective perceptions in cases of vertigo. The definition of a diagnosis (according to ICD-10) is based on reference ranges and is medical standard.

## Notes

### Competing interests

The author declares that he has no competing interests.

## Figures and Tables

**Table 1:**
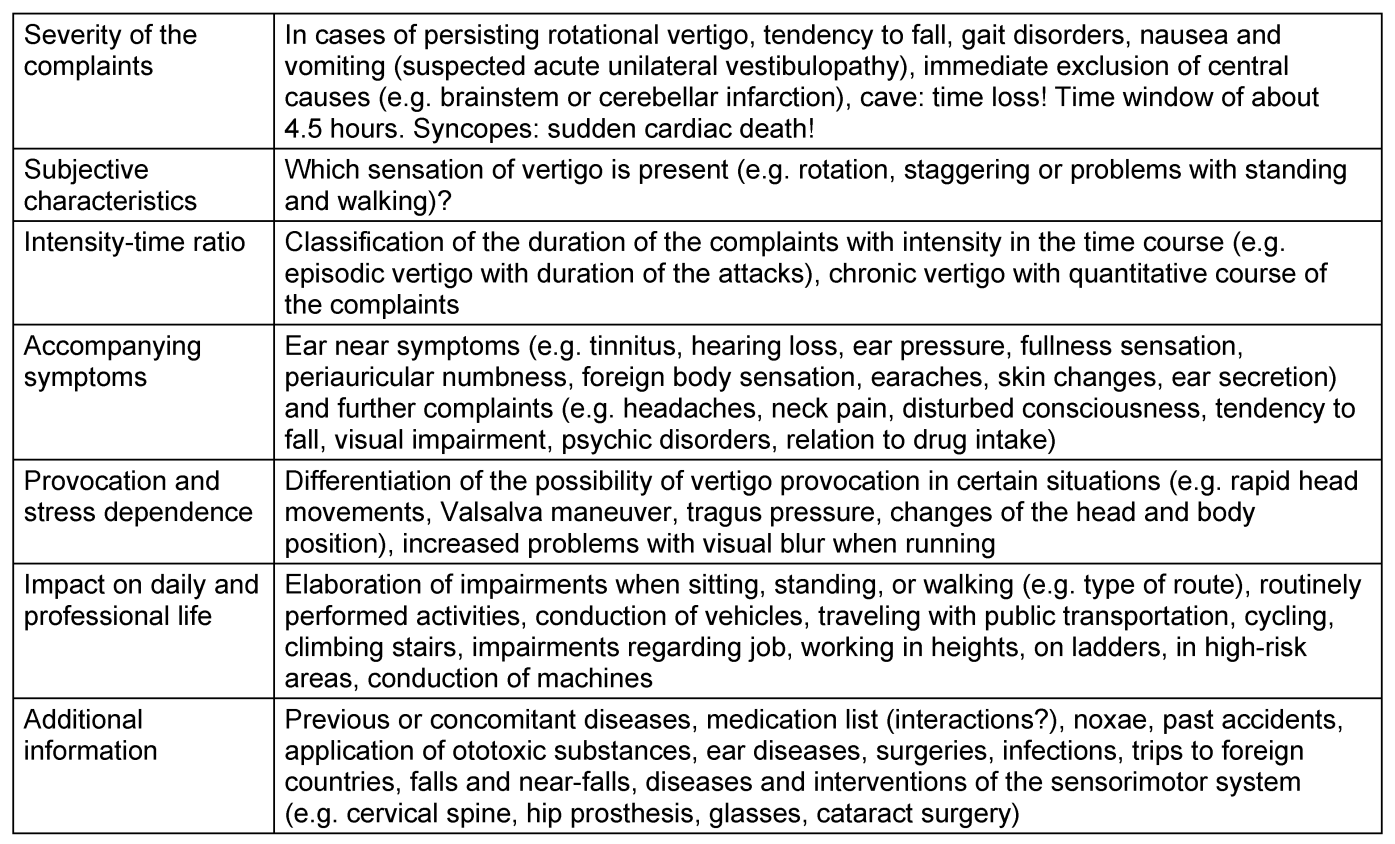
Structured anamnesis for vertigo. The priority question is the severity of the complaints (modified according to [94])

**Table 2:**
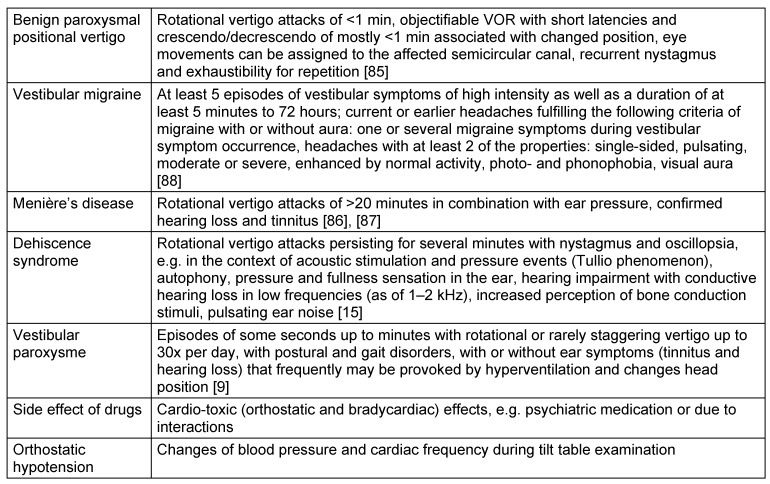
Anamnestic differentiation of episodic vertigo (modified according to [94])

**Table 3:**
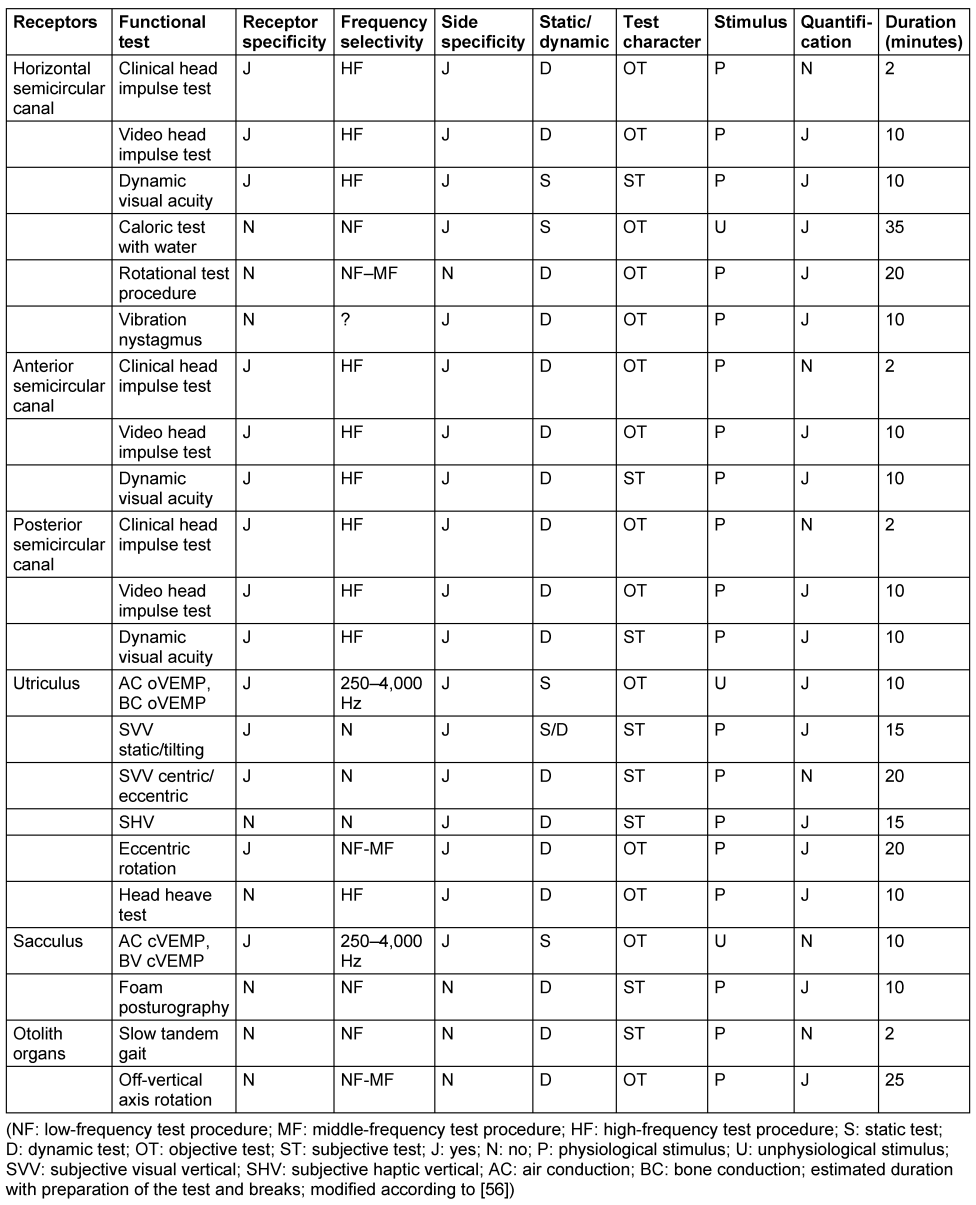
Functional tests for the 5 labyrinthine receptors and reflective interrelations
